# Hospital Meetings

**Published:** 1906-01-27

**Authors:** 


### HOSPITAL MEETINGS.

### THE ROYAL WATERLOO HOSPITAL FOR CHILDREN AND WOMEN.

The ninetieth annual Court of the Governors of this hospital was held at the Mansion House on January 19, the Lord Mayor being in the chair.

The Lord Mayor, in moving that the Royal Waterloo Hospital for Children and Women was in every way worthy of support, and had strong and distinct claims upon the City of London, said that the hospital had been born in the City, and was the oldest institution for the treatment of children's diseases. Though its title had been altered in late years, it still continued to give a large amount of attention to the diseases of the young. The old hospital having been condemned as insanitary in 1902, the present handsome and commodious building had been erected, and was now in occupation. The cost of the new building, exclusive of site, but inclusive of furniture and apparatus, had been £35,000, of which about £20,000 had been received and £5,000 had been promised conditionally. Special efforts were made during the past year to extinguish the building debt, etc., and splendid work in this direction had been done by the Ladies' Association. A monster bazaar had been arranged to take place next June by this association, which had already been successful in obtaining large sums of money. The Special Appeal Committee had also done great service, and they were in hopes that by the end of the year the debt on the building fund would be paid off. The present building only consisted of two-thirds of the proposed hospital, the remaining third must wait until they had the necessary funds in hand. He appealed to them all to do what they could to raise money for the payment of the debt on the building fund, and to try and obtain additional annual subscribers.

The resolution was seconded by Mr. W. Seymour Eastwood, who enlarged on the excellent work done by the hospital in the poorest districts, and said that great pressure had been put upon them to open more wards. He appealed to the public to help by increased annual subscriptions, and so to enable the hospital to give the poor people the benefits they so urgently needed.

Mr. John Topham Richardson (Treasurer) moved the adoption of the report and accounts, and also a resolution stating that as the present income was quite insufficient for the maintenance of the new hospital, and the general fund and building fund were both largely overdrawn, there was a most urgent need for new annual subscriptions and for donations to the building fund.

The resolutions having been seconded and carried, a vote of thanks to the Board of Governors, honorary medical staff, and other officers of the hospital was passed.

The election of officers for the ensuing year having been effected, the proceedings terminated with the usual vote of thanks to the Lord Mayor for taking the chair.

